# Potential application of foveal structural measurements in treatment decision for retinopathy of prematurity: an OCT-based study

**DOI:** 10.1186/s40942-023-00499-0

**Published:** 2023-10-18

**Authors:** Fatemeh Bazvand, Farhad Salari, Navid Hasani Barzi, Fariba Ghassemi, Anahid Maleki, Afsar Dastjani Farahani, Marjan Imani Fooladi, Naeeme Taslimi Taleghani, Maryam Khoshnoud Shariati, C. Armitage Harper, Mohammadreza Mehrabi Bahar

**Affiliations:** 1 Farabi Eye Hospital, Tehran University of Medical Sciences, Retina services, Tehran, Iran; 2 Department of Anesthesiology and Critical Care, Tehran University of Medical Sciences, Children Medical Center, Tehran, Iran; 3 Clinical Research Development Center, Mahdiyeh Educational Hospital, Shahid Beheshti University of Medical Sciences, Tehran, Iran; 4 Austin Retina Associates, University of Texas-San Antonio, San Antonio, USA

## Abstract

**Purpose:**

To investigate foveal changes in infants with ROP not requiring treatment(nROP) and ROP infants needing treatment (tROP) using a handheld SD-OCT device.

**Method:**

We performed horizontal SD-OCT scans through the fovea in 156 eyes of 81 infants diagnosed with ROP. Foveal immaturity indices including the presence of inner retinal layers (IRL), absence of foveal outer nuclear layers widening (ONL) and attenuation of hyperreflective outer segment layers (OS), presence and type of cystoid macular edema (CME), epiretinal membrane (ERM), foveal pit depth (FPD), foveal pit width (FPW) and central foveal thickness (CFT) were calculated. The multivariate logistic regression model was used to predict the need for treatment based on OCT measurements.

**Results:**

The shape of the foveolar pit was not significantly different among tROP and nROP groups (P-value = 0.287, Chi-square test). IRL extrusion was incomplete in both tROP and nROP groups (P-value = 0.0.41, Chi-square test). Nevertheless, the presence of thicker IRL was more frequent in the nROP group in comparison with the tROP group (100% vs.64.8%, P-value = 0.001). CME was observed in 29% of eyes in the tROP group and 40% of eyes in the nROP group; however, this difference was not statistically significant (P-value = 0.32, Chi-square test). ERM was detected in 15 (75%) and 84 (65.6%) eyes in the nROP and tROP groups, respectively (P-value = 0.39, Chi-square test). Multivariate logistic regression analyses showed that the need for treatment was significantly associated with gestational age (GA), CFT and FPD (P-values 0.001 and 0.002 respectively).

**Conclusions:**

This study demonstrated GA, foveal pit depth and the central foveal thickness could accurately predict the need for treatment with sensitivity, specificity, and diagnostic accuracy of 97%, 65% and 91.7% respectively.

## Introduction

Normal foveal development consists of staged specialization of both inner and outer retinal layers. The foveal excavation formation begins at 25 weeks gestational age and foveal vascular and structural maturation continues up to 4 years after birth [1]. Several studies on premature infants have demonstrated arrest of foveation compared to term infants and even children with a history of prematurity have shown signs of macular development arrest including maldeveloped foveal excavation and preserved inner retinal layers in foveola [2–5]. Retinopathy of prematurity (ROP) is a potentially blinding vasoproliferative retinal disease affecting extremely premature infants [6]. In the current practice, ophthalmologists focus on ophthalmoscopic examination to diagnose and manage ROP [7, 8]. However, long-term follow-up of patients with a history of ROP treatment has revealed a wide range of visual outcomes that couldn’t be attributed solely to vascular grading [9]. With the advent of handheld optical coherence tomography (OCT), recent studies have evaluated the structural changes in ROP patients to find the role of foveal morphology in final visual function. For instance, it has been shown that a thinner foveal outer nuclear layer is associated with retinal functional loss in ROP patients [5]. In addition OCT-based based studies revealed structural changes in severe ROP patients in comparison with milder spontaneously regressed forms including thicker central foveal thickness, malformed foveal pit, presence of cystoid macular edema (CME), and epiretinal membrane [10, 11]. In addition, ROP patients treated with laser photocoagulation or anti-VEGF show a different macular structure later in life, which is unsettled that this difference is due to the treatment itself or toted with ROP severity [12, 13].

This study sought to measure and characterize the structural findings in pre-treatment macular OCT of patients with ROP and to develop a predicting model to utilize foveal measurements for the determination of the need for treatment.

## Method

### Subjects

This cross-sectional study was conducted between 2019 and 2021 in Farabi Eye Hospital with approval from the local ethics committee of the Tehran University of Medical Sciences (https://ethics.research.ac.ir/IR.TUMS.FARABIH.REC.1400.065) and is complies with the tenets of the Declaration of Helsinki. Informed consent was obtained from all the involved infants’ parents or legal guardians. We included 156 eyes of 81 neonates with ophthalmoscopic diagnosis of retinopathy of prematurity by ROP experts from Farabi Eye Hospital. Gestational age (GA), birth weight (BW), age at examination (postmenstrual age (PMA)), and major medical problems such as intraventricular hemorrhage and sepsis were collected from their neonatologists.

The infants were classified into two groups: The preterm infants with type 1 ROP who were scheduled for receiving treatment by an ROP expert (treatment group (tROP)) [14] and the second group includes infants without ROP or with ROP who don’t require treatment (no treatment group(nROP). The tROP comprises two subgroups: infants who were going to receive laser photocoagulation(ltROP) and neonates who were going to receive intravitreal bevacizumab (btROP). Generally, ROP in zone I or posterior zone II was included in the btROP subgroup, and more anterior involvement was included in the ltROP subgroup based on ROP expert opinion. All the subjects were examined using Handheld portable SD-OCT (Optovue iVue SD-OCT Wellness report; Optovue Corporation, Fremont, CA) after pupil dilatation and before treatment, and under general anesthesia or sedation. The imaging was performed in the same visit that the diagnosis of ROP made by the expert. Some preterm infants were examined in the neonatal intensive care unit (NICU) and routinely they received sedation for fundus examination with sclera depression for controlling stress and pain in addition to topical anesthesia. Intravitreal injections were performed after administration of 10% povidone-iodine for periocular skin, and 5% povidone-iodine for ocular surface, half of the adult doses of bevacizumab (0.625 mg/0.025 mL) were injected with a 30-gauge needle 1-1.5 mm behind the limbus into the vitreous cavity. Topical gentamycin or sulfacetamide was given for 3 days post-injection. In cases that need laser ablation, the indirect laser was performed in avascular areas using a confluent or near-confluent pattern with moderate intensity after general anesthesia and full pupillary dilation. Topical gentamycin or sulfacetamide, topical mydrax 0.5% and topical betamethasone were prescribed for 7 days in patients receiving indirect laser.

To control imaging artifacts, the examination without any sedation or general anesthesia and images with low quality were excluded. Patients with unstable general conditions or hazy media such as corneal edema were also excluded. Infants’ ophthalmoscopic ROP staging, zone, and presence of plus were documented by an ROP expert. Of 156 eyes, 133 required treatments (tROP group). Twenty-two eyes required Bevacizumab injection(btROP) and the other 111 eyes received indirect laser photocoagulation (ltROP).

### Image processing

**Fig. 1:**
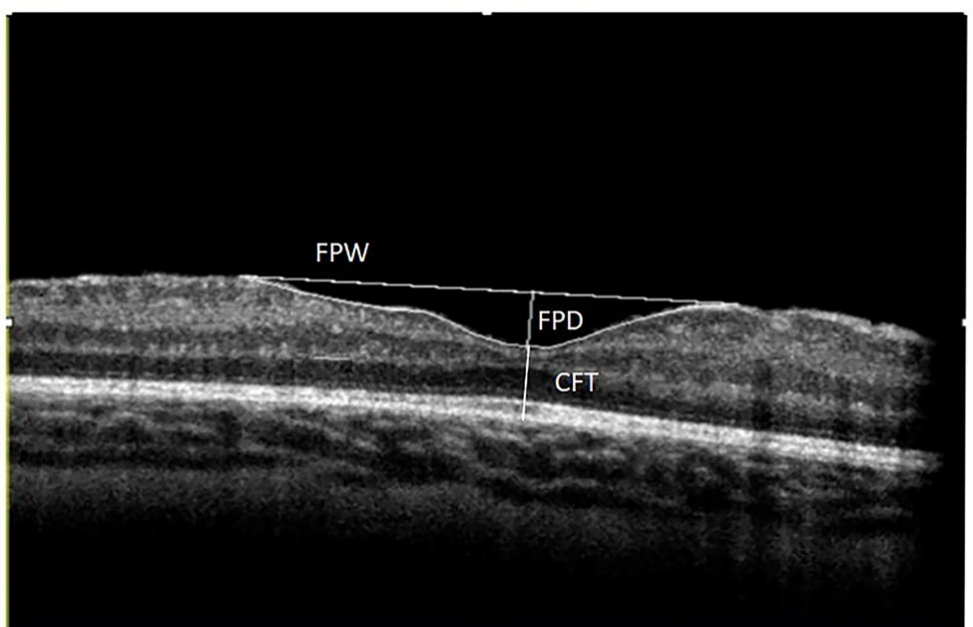
This picture shows foveal measurements in sample OCT of an ROP patient. FPA equals the rectangular surface area that covers the foveal contour and the line connecting two parafoveal peaks (FPW). Abbreviations: CFT, central foveal thickness; FPD, foveal pit depth; FPW, foveal pit width; FPA, foveal pit area

OCT scans were obtained by a single experienced examiner (FB). Foveal measurements were performed on the horizontal B-scan with the steepest foveal excavation in which adjacent B-scans showed a shallower excavation. The distance between the internal limiting membrane and the retina pigment epithelium at the steepest point of the foveal pit was defined as Central foveal thickness (CFT). The distance between two parafoveal peaks was considered as the foveal pit width (FPW) and by drawing a perpendicular line from FPW crossing the steepest point of the foveal pit, we measured the foveal pit depth (FPD). The area between FPW and the foveal pit contour was calculated and represented as the foveal pit area (FPA) (Fig. 1). All of the foveal parameters were measured by an experienced co-author (MRMB) using Image J analysis software. FPD, FPW and FPA were only calculated in eyes with discernible foveal pit (eyes with cystoid macular edema or epiretinal membrane plus cystoid macular edema were excluded). However, in cases of ILM thickening or epiretinal membrane with detectable foveal pit, measurements were performed according to above-mentioned protocol.

**Fig. 2:**
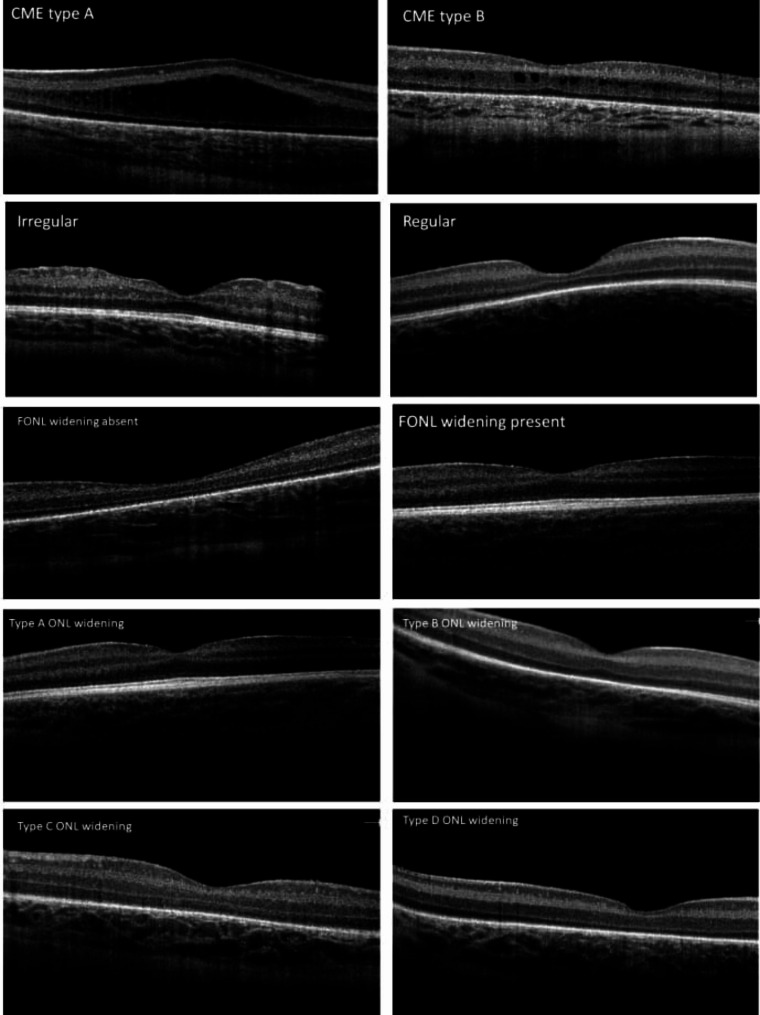
Morphologic characteristics of foveal immaturity observed by use of spectral-domain optical coherence tomography (SD-OCT). The first line shows two phenotypes of cystoid macular edema; A, the presence of vertical edema and complete splitting of retinal layers without apparent foveal pit and B, the presence of cystic and vacuoles in retinal layers with foveal pitting. The second line illustrated a sample of foveal irregularity in ROP patients. The third line depicts samples for the presence or absence of Outer nuclear layer widening in ROP patients. The fourth and fifth lines are showing four classes of ONL widening: type A, ONL thickness increases from the periphery toward the fovea; type B, ONL thickness reduces or remains stable from the periphery toward the parafovea and then increases at the fovea; type C: no change in ONL thickness from the periphery toward foveal center, and type D, ONL thickness reduce from the periphery toward the fovea

**Fig. 3:**
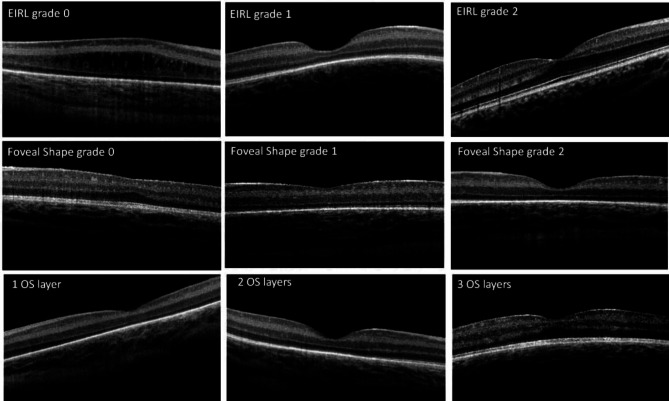
Morphologic characteristics of foveal immaturity observed by use of spectral-domain optical coherence tomography (SD-OCT). The first line displays the three grades of inner retinal layers’ extrusion from the foveal center: grade 1, incomplete INL extrusion, and grade 0; absent of INL extrusion. The second line shows the three grades of foveal shape maturity: grade 0, absence of foveal pit, grade 1 shallow pit, and grade 2 normal deep foveal pit. The last line shows the types of outer segment layers in ROP patients: All of the external limiting membrane (ELM), Ellipsoid zone (EZ) and RPE are detectable, only 2 of them are visible and only 1 hyper-reflective layer was visible

To discern the stage of foveal maturation in ROP patients, the presence of cystoid macular edema(CME) and its type, epiretinal membrane (ERM), hyperreflective foci (HRF), the shape of the foveal pit, regularity of foveal pit, extrusion of inner retinal layers (EIRL) from the fovea, Thickness of foveal inner retinal layers (FIRL), foveal outer nuclear layer (FONL) widening, type of FONL widening and number of visible outer segment layers in fovea were evaluated and described by two experienced ROP experts (FB and MRMB). Two co-authors evaluated all patients’ images separately and cases of discrepancy were reevaluated and discussed in a third session. CME was categorized into 2 types regarding Vinekar’s classification [15]: A, the presence of vertical edema and complete splitting of retinal layers without apparent foveal pit and B, the presence of cystic and vacuoles in retinal layers with foveal pitting (Fig. 2). Extrusion of inner retinal layers (EIRL) was classified into 3 grades according to Thomas’s classification [16]; grade 2, normal fovea with complete inner nuclear layer (INL) extrusion from central fovea; grade 1, incomplete INL extrusion and grade 0; absent of INL extrusion (Fig. 3). The shape of the foveal pit was categorized into 3 grades: grade 0, absence of the foveal pit, grade 1 shallow pit and grade 2 normal deep foveal pit (Fig. 3). The foveal outer nuclear widening was defined as the presence of ONL lengthening in comparison with the parafoveal area (Fig. 2). In addition, FONL widening was classified into 4 groups: type A, ONL thickness increases from the periphery toward the fovea; type B, ONL thickness reduces or remains stable from the periphery toward the parafovea and then increases at the fovea; type C: no change in ONL thickness from the periphery toward foveal center, and type D, ONL thickness reduce from the periphery toward fovea (Fig. 2). Also, the number of visible hyperreflective layers in the foveal outer segment was calculated: 3; All of the external limiting membrane (ELM), Ellipsoid zone (EZ) and RPE are detectable;2, only 2 of them are visible and 1, only 1 hyper-reflectivetive layer was visible (Fig. 3).

### Statistical analysis

We described Retinal parameters using mean (standard deviation), and median (interquartile range). We used the Pearson correlation test to assess the correlation of the baseline characteristics such as BW and GA with foveal measurements. In the comparison of treatment type and no need for treatment, multiple comparisons were considered by Sidak’s method. To obtain a parsimonious score that can predict the need for treatment we used a logistic regression with a backward selection method. The diagnostic ability of this score was investigated by the ROC curve and its area under the curve (AUC). We used Youden’s index to obtain the best cutoff point. In addition, sensitivity, specificity, and diagnostic accuracy were reported. All statistical analysis performed by SPSS (IBM Corp. Released 2017. IBM SPSS Statistics for Windows, Version 25.0. Armonk, NY: IBM Corp).

## Results

Within the study period (from December 2019 to March 2022), 156 eyes from 81 infants with ROP met the criteria to be included in this study. There were 40 (48.7%) male and 41 (50.6%) female patients. The mean gestational age was 29.57 ± 2.58 weeks (median 29, interquartile range: 28–31). The mean birth weight of the infants was 1350 ± 461 g (range: 650–3100). Based on inclusion criteria, 21 eyes did not need any treatment (nROP), and 135 eyes required treatment (tROP group), of which 22 and 113 received IVB (btROP)and laser photocoagulation treatment (ltROP), respectively. All patients’ baseline demographic and clinical characteristics are listed in Table 1. There wasn’t a significant difference between tROP and nROP regarding major medical problems such as intraventricular hemorrhage and sepsis (all P-values > 0.05, Chi-square test) (Table 1).

**Table 1:** Baseline characteristics of patients included in the study

		Need to treat			Treatment		
		No	Yes	Sig.	IVB	Laser	Sig.
Variable	Label						
Sex	Male	7(33.3%)	69(51.1%)	0.35	11(50.0%)	58(51.3%)	0.84
	Female	14(66.7%)	66(48.9%)		11(50.0%)	55(48.7%)	
Laterality	OD	10(47.6%)	68(50.4%)	0.48	11(50.0%)	57(50.4%)	0.02
	OS	11(52.4%)	67(49.6%)		11(50.0%)	56(49.6%)	
Gestational Age	mean ± SD	31 ± 5	29 ± 2	0.12	28 ± 2	30 ± 2	0.001
	median (Q1,Q3)	29(28,35)	29(28,31)		28(26,29)	30(28,31)	
Birth Weight	mean ± SD	1579 ± 661	1303 ± 408	0.052	1131 ± 236	1339 ± 427	0.041
	median (Q1,Q3)	1500(1100,2010)	1195(1040,1600)		1100(970,1270)	1200(1050,1600)	
Treatment with O2	No	20(95.2%)	22(16.3%)	0.001	4(18.2%)	18(15.9%)	0.75
	Yes	1(4.8%)	113(83.7%)		18(81.8%)	95(84.1%)	
Mechanical Ventilation	No	20(95.2%)	75(55.6%)	0.039	7(31.8%)	68(60.2%)	0.13
	Yes	1(4.8%)	60(44.4%)		15(68.2%)	45(39.8%)	
Sepsis	No	21(100.0%)	127(94.1%)	0.41	20(90.9%)	107(94.7%)	0.73
	Yes	0(0.0%)	8(5.9%)		2(9.1%)	6(5.3%)	
Hydrocephaly	No	21(100.0%)	133(98.5%)	0.69	22(100.0%)	111(98.2%)	0.63
	Yes	0(0.0%)	2(1.5%)		0(0.0%)	2(1.8%)	
Jaundice	No	20(95.2%)	82(60.7%)	0.07	11(50.0%)	71(62.8%)	0.38
	Yes	1(4.8%)	53(39.3%)		11(50.0%)	42(37.2%)	
Intra Ventricular Hemorrhage	No	21(100.0%)	135(100.0%)		22(100.0%)	113(100.0%)	
	Yes	0(0.0%)	0(0.0%)		0(0.0%)	0(0.0%)	
Blood transfusion	No	21(100.0%)	77(57.0%)	0.008	12(54.5%)	65(57.5%)	0.81
	Yes	0(0.0%)	58(43.0%)		10(45.5%)	48(42.5%)	
Thrombocytopenia	No	21(100.0%)	135(100.0%)		22(100.0%)	113(100.0%)	
	Yes	0(0.0%)	0(0.0%)		0(0.0%)	0(0.0%)	
Platelet transfusion	No	21(100.0%)	134(99.3%)		21(95.5%)	113(100.0%)	
	Yes	0(0.0%)	1(0.7%)		1(4.5%)	0(0.0%)	
Anemia	No	21(100.0%)	111(82.2%)	0.15	19(86.4%)	92(81.4%)	0.37
	Yes	0(0.0%)	24(17.8%)		3(13.6%)	21(18.6%)	
Gestational Diabetes Mellitus	No	21(100.0%)	115(85.2%)	0.18	20(90.9%)	95(84.1%)	0.45
	Yes	0(0.0%)	20(14.8%)		2(9.1%)	18(15.9%)	
COVID	No	21(100.0%)	133(98.5%)	0.69	22(100.0%)	111(98.2%)	0.63
	Yes	0(0.0%)	2(1.5%)		0(0.0%)	2(1.8%)	

### OCT Findings

Detailed findings of macular SD-OCT in each study group are represented in Table 2. ERM was detected in 15 (75%) and 84 (65.6%) eyes in the nROP and tROP groups, respectively (P-value = 0.39, Chi-square test). Also, the percentage of ERM was not significantly different among ltROP and btROP subgroups (P-value = 0.82, Chi-square test). CME was observed in 29% of eyes in the tROP group and 40% of eyes in the nROP group; however, this difference was not statistically significant (P-value = 0.32, Chi-square test). The type of CME was also not significant between groups.

**Table 2:** Morphology findings and measurements of fovea by using optical coherence tomography in premature patients

		No	Yes	Sig.	IVB	Laser	Sig.
Variable	Label						
ERM	No	5(25.0%)	43(34.7%)	0.394	7(36.8%)	36(34.3%)	0.829
	Yes	15(75.0%)	81(65.3%)		12(63.2%)	69(65.7%)	
CME	No	12(60.0%)	88(71.0%)	0.323	17(89.5%)	71(67.6%)	0.053
	Yes	8(40.0%)	36(29.0%)		2(10.5%)	34(32.4%)	
CME type	A	6(75.0%)	22(48.6%)	0.322	2(100.0%)	20(60.7%)	0.325
	B	2(25.0%)	13(37.1%)		0(0.0%)	13(39.4%)	
Foveal Pit shape	No	7(35.0%)	24(19.4%)	0.287	3(15.8%)	21(20.0%)	0.003
	Shallow pit	2(10.0%)	16(12.9%)		7(36.8%)	9(8.6%)	
	Well formed pit	11(55.0%)	84(67.7%)		9(47.4%)	75(71.4%)	
Foveal pit irregularity	Irregular	0(0.0%)	3(2.9%)	0.501	1(6.3%)	2(2.3%)	0.394
	Regular	15(100.0%)	99(97.1%)		15(93.8%)	84(97.7%)	
EIRL	Complete	0(0.0%)	0(0.0%)	0.417	0(0.0%)	0(0.0%)	0.618
	Incomplete	0(0.0%)	4(3.2%)		1(5.0%)	3(2.9%)	
	Absent	20(100.0%)	121(96.8%)		19(95.0%)	102(97.1%)	
FIRL thickness	Thick	20(100.0%)	81(64.8%)	0.001	8(40.0%)	73(69.5%)	0.011
	Thin	0(0.0%)	44(35.2%)		12(60.0%)	32(30.5%)	
FONL widening	Absent	20(100.0%)	111(88.8%)	0.115	16(80.0%)	95(90.5%)	0.173
	Present	0(0.0%)	14(11.2%)		4(20.0%)	10(9.5%)	
ONL widening type	A	0(0.0%)	6(4.8%)	0.057	3(15.0%)	3(2.9%)	0.001
	B	0(0.0%)	21(16.8%)		8(40.0%)	13(12.4%)	
	C	4(20.0%)	35(28.0%)		3(15.0%)	32(30.5%)	
	D	16(80.0%)	63(50.4%)		6(30.0%)	57(54.3%)	
FOS layers	1	19(95.0%)	66(53.2%)	0.002	6(30.0%)	60(57.7%)	< 0.001
	2	1(5.0%)	35(28.2%)		3(15.0%)	32(30.8%)	
	3	0(0.0%)	23(18.5%)		11(55.0%)	12(11.5%)	
HRF	No	17(85.0%)	103(82.4%)	0.775	16(80.0%)	87(82.9%)	0.758
	Yes	3(15.0%)	22(17.6%)		4(20.0%)	18(17.1%)	
CFT	mean ± SD	188 ± 77.6	217.09 ± 105.9	0.24	237.21 ± 80.5	214.1 ± 109.3	0.09
	median (Q1,Q3)	160 (135,265)	179 (145,251)		210 (190,310)	170 (140,250)	
FPD	mean ± SD	934.79 ± 376.6	1057.42 ± 332.4	0.06	783.5 ± 273.2	1098.92 ± 322.3	0.003
	median (Q1,Q3)	797 (760,1020)	1071 (830,1265)		675 (578,1082)	1102 (871,1273)	
FPW	mean ± SD	70.79 ± 46.1	103.83 ± 70	0.015	72.5 ± 44.2	108.58 ± 72.2	0.022
	median (Q1,Q3)	68 (31,82)	96 (71,129)		67 (32,81)	98 (81,131)	
FPA	mean ± SD	41.5 ± 44.8	66.53 ± 99.5	0.041	28 ± 24.03	72.36 ± 105.3	0.006
	median (Q1,Q3)	29 (17,37)	48 (26,68)		20 (9,33)	51 (30,69)	

ERM; epiretinal membrane, CME; cystoid macular edema, HRF; hyperreflective foci, EIRL; extrusion of inner retinal layers, FIRL; foveal inner retinal layers, FONL; foveal outer nuclear layer, ONL; outer nuclear layer, CFT; central foveal thickness, FPD; foveal pit depth, FPW; foveal pit width, FPA; foveal pit area

The shape of the foveolar pit was not significantly different among tROP and nROP groups (P-value = 0.287, Chi-square test), however immature foveolar pit was more evident in btROP than ltROP subgroup (p-value = 0.003, Chi-square test). There wasn’t any significant difference between the irregularity of the foveal contour between tROP and nROP groups (P value = 0.5, Chi-square test).

IRL extrusion was incomplete in both tROP and nROP groups (P-value = 0.0.41, Chi-square test). Nevertheless, the presence of thicker IRL was more frequent the in nROP group in comparison with the tROP group (100% vs.64.8%, P-value = 0.001). Also, persistent thick IRL was more prevalent in ltROP than btROP groups (69.5% vs. 40%, P-value = 0.01).

In contrast to 14 eyes (11.2%) in tROP, none of the eyes in the nROP group had OS widening (P-value = 0.1, Chi-square test). In the treatment group also there wasn’t any significant difference between the rate of ONL widening eyes of ltROP and btROP subgroups. However, the type of ONL widening was dissimilar among treatment subgroups (p-value = 0.001, Chi-square). Type A and B ONL widening was higher in btROP in comparison to ltROP (P-value = 0.02 and 0.002, respectively, Chi-square test). Type D ONL widening was more frequently observed in ltROP than btROP (P-value = 0.046, Chi-square test).

None of the eyes in the nROP group showed to have all 3 outer segments layers, 1 layer was observed in 95% and 2 layers in 5% of eyes, whereas in tROP 18.5% had all 3 OS layers, 28.2% had 2 layers and 53.2% had only 1 layer. This difference was significant (P-value = 0.002, Chi-square). Among treatment subgroups, a well-formed outer segment was significantly more evident in btROP than ltROP (P < 0.001, Chi-square). HRF was observed in 3 (15%) and 22(17.1%) eyes in nROP and tROP groups, respectively but the percentage was not statistically significant.

Central foveal measurements including foveal pit depth (FPD), foveal pit width (FPW), foveal pit area (FPA), and central foveal thickness (CFT) are shown in Table 2. Although mean CFT was higher in tROP than nROP, the difference didn’t reach statistical significance (217.09 ± 105.9, 188 ± 77.56 in tROP and n ROP respectively, P value = 0.32, Mann Whitney U test). FPD and FPA were significantly higher in tROP than nROP (P-value < 0.05, Mann Whitney U test). In addition, in comparison between ltROP and btROP, all FPW, FPA and FPD were significantly higher in eyes with ltROP than btROP.

Correlation between GA, PMA, BW, and foveal measurements.

Although postmenstrual age (PMA) was not correlated with foveal measurements, there was a moderately inverse correlation between gestational age (GA) and CFT (r =-0.39, P-value < 0.001, Pearson correlation test) (Table 3). In addition, GA was positively correlated with FPW and FPA (P-value < 0.05, Table 3). Birth weight was also negatively correlated with CFT and weakly correlated with FPW and FPD (all P-values < 0.05, Pearson correlation test).

**Table 3:** Correlation between the baseline characteristics of patients and foveal measurements

		Central foveal thickness	Foveal pit width	Foveal pit Depth	Foveal pit area
GA	Pearson Correlation	-0.395	0.303	0.206626361	0.257
	Sig. (2-tailed)	< 0.001	0.006	0.06	0.019
BW	Pearson Correlation	-0.358	0.256	0.233	0.198
	Sig. (2-tailed)	< 0.001	0.02	0.03	0.07
PMA	Pearson Correlation	0.166	-0.223	-0.193	-0.119
	Sig. (2-tailed)	0.109	0.081	0.132	0.353

GA; gestational age, BW; birth weight, PMA; postmenstrual age

**Fig. 4:**
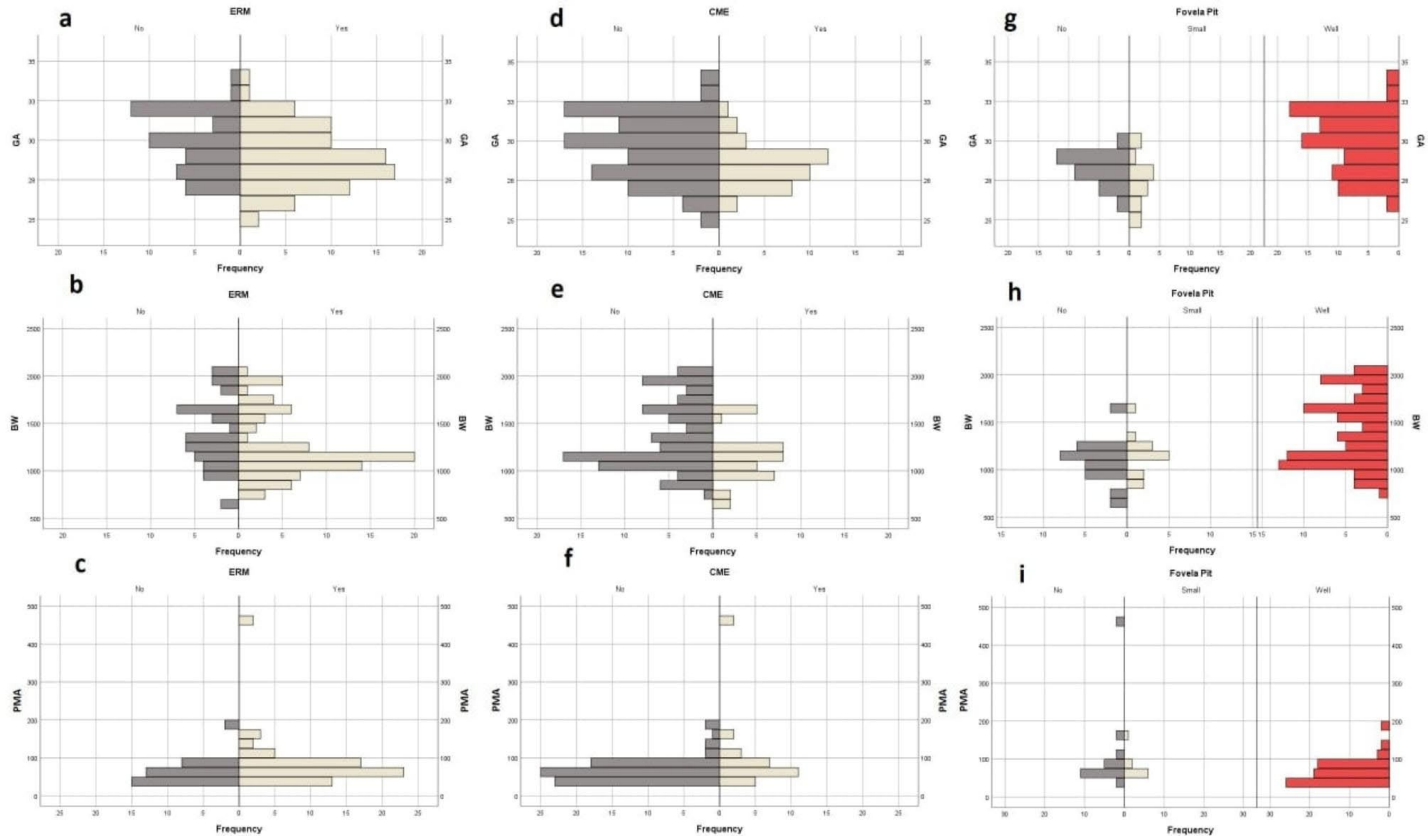
This figure shows the difference in baseline characteristics of ROP patients with and without foveal findings. The first column (a, b, and c) compares the mean GA, BW, and PMA of patients who were found to have ERM in their macular OCT and patients who didn’t have ERM. The second column (d, e, and f) compares the mean GA, BW, and PMA of patients who had or did not have CME in their macular OCT. The last column (g, h, and i) compares the mean GA, BW, and PMA between three types of foveal shape immaturity observed in ROP patients

Eyes with ERM had significantly lower GA and BW (P values were 0.024 and 0.027 respectively, Mann-Whitney U test) (Fig. 4a, b). However, the difference in the mean PMA was insignificant between eyes with and without ERM (P-value = 0.07, Mann-Whitney U test) (Fig. 4c). Similarly, eyes with CME had significantly lower GA and BW (P values were 0.003 and 0.011 respectively, Mann-Whitney U test) (Fig. 4d, e). Whereas, the difference in the mean PMA was insignificant between eyes with and without CME (Fig. 4f). Additionally, eyes with well-formed foveal pit had significantly higher GA and BW (P-values < 0.001, Kruskal Wallis test) (Fig. 4g, h); however, there wasn’t a significant difference between PMA of eyes with well-formed foveal pit and immature forms (P-value = 0.059, Kruskal Wallis test) (Fig. 4i).

### Regression model

To explore the relationship between foveal measurements and the need for treatment multivariate logistic regression model was used. Table 4 shows the result of multivariate analysis for the determination of the need for treatment based on GA, BW, FPD, FPA, FPW and CFT (Table 4). The significant parameters were GA, CFT and FPD. With GA, CFT and FPD in the logistic regression model, the mathematical equation for predicting the need for treatment is as follows:

**Table 4:** Stepwise multivariate logistic regression analysis with backward methodfor the factors correlated with treatment needs in ROP patients.

	B	S.E.	Wald	df	Sig.	Exp(B)	95% C.I.for EXP(B)	
							Lower	Upper
CFT	0.050	0.020	7.820	1.000	0.010	1.050	1.020	1.090
FPD	0.080	0.020	10.940	1.000	0.000	1.080	1.030	1.130
GA	-0.460	0.180	6.340	1.000	0.010	0.630	0.440	0.900
Constant	0.800	5.760	0.020	1.000	0.890	2.240		
Variables not in the equation:								
FPW	0.000	0.000	0.000	1.000	0.970	1.000	1.000	1.000
FPA	0.000	0.010	0.020	1.000	0.880	1.000	0.990	1.010
BW	-0.370	0.210	2.960	1.000	0.090	0.690	0.450	1.050

CFT; central foveal thickness, FPW; foveal pit width, FPD; foveal pit depth, FPA; foveal pit area; BW, birth weight; GA, gestational age

$$\varvec{P}\varvec{r}\varvec{e}\varvec{d}\varvec{i}\varvec{c}\varvec{t}\varvec{i}\varvec{o}\varvec{n} \varvec{s}\varvec{c}\varvec{o}\varvec{r}\varvec{e}=\varvec{C}\varvec{F}\varvec{T}\times 0.05+\varvec{F}\varvec{P}\varvec{D}\times 0.08-0.46\times \varvec{G}\varvec{A}+0.8$$

**Fig. 5:**
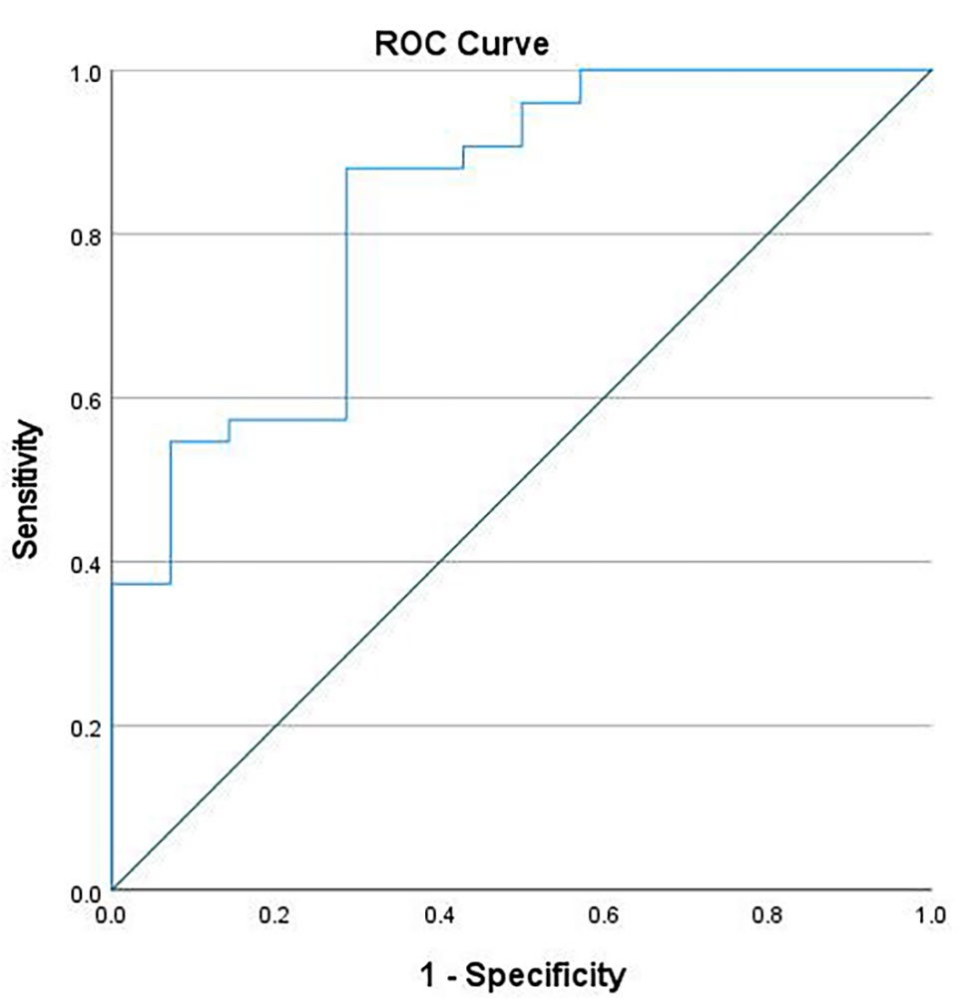
The receiver operating curve is shown for the multivariate logistic regression model for predicting treatment needs based on retinal findings

To assess the diagnostic accuracy of the prediction score, we derived the receiver operating curve and AUC (Fig. 5). The AUC of the model was 0.89. The maximum cut-off value for prediction score based on Youden-index was 0.622 and with this cut-off, the model showed to have sensitivity, specificity and diagnosis accuracy of 97%, 65% and 91.7% respectively.

## Discussion

Foveal development is a complicated and lengthy process starting at 25 weeks of fetal age and continuing through adulthood [16]. Foveal maturation is characterized by pit formation due to centrifugal migration of inner retinal layers and an increase in central outer nuclear layer thickness due to centripetal migration of cone photoreceptors [1, 17–21]. Several studies have documented foveal development arrest in patients with a history of retinopathy of prematurity (ROP) even in subjects whose disease spontaneously regressed [22, 23]. This study characterized different OCT-based findings of foveal development arrest in ROP patients who require treatment(tROP) and compared them with ROP patients who didn’t require treatment (nROP). We also compared pretreatment OCT of patients undergoing laser photocoagulation (LPC) (ltROP) with ROP patients requiring intravitreal bevacizumab (IVB) (btROP), to find a possible utility of OCT imaging in the improvement of ROP management.

One of the features of the developing fovea is the presence of CME or ERM. The rate of CME in this case series was 44% which was similar to previous reports [11, 22]. We also found that eyes with CME had significantly lower GA and BW (Fig. 4). However, the presence of CME or the type of CME couldn’t distinguish the need for treatment in ROP patients (Table 2). Similar to our study, Gursoy et al. didn’t report the difference in the rate of CME and ERM in the evaluation of 15 ROP patients without treatment indication and 21 thresholds ROP [10]. Dysregulation of VEGF has been proposed as a main mechanism of CME formation in ROP patients as the presence of CME is correlated to the severity of ROP [11, 23]. Nevertheless, due to the rarity of OCT application in the diagnosis and management of ROP infants, many untreated ROP infants had cystoid macular changes (in this case series 6 ROP patients (30%) showed to have type A CME (presence of vertical edema and complete splitting of retinal layers without apparent foveal pit)). The significance of these findings and their link to the final visual outcomes of untreated patients should be studied in future investigations. We also showed there wasn’t a significant difference between the rate and type of CME in patients treated with LPC or IVB. As the decision for modality of treatment is mainly based on the zone of involvement not the amount of VEGF release, this lack of difference could be explained. However, the application of CME and its type of modification of treatment should be investigated in the future.

We also found that eyes with ERM had significantly lower GA and BW (Fig. 4). In this study, ERM was a frequent finding as 66% of infants with ROP had ERM. In addition, ERM couldn’t differentiate the need for treatment in ROP patients (Table 2). Results of this study also revealed that there wasn’t a significant difference between the rate of ERM formation in patients treated with LPC or IVB. Whereas in a previous study by Gursoy et al., with similar GA and BW, ERM was presented in only 5% of patients. In adult studies, it is proven that the presence of ERM could be affected by ethnicity and depend on comorbidities. Therefore, the observed difference could be attributed to the dissimilarity of ethnicity and coexisting medical problems.

We found that in contrast to the inner retinal layers, the outer layers of the retina are generally better formed in the treatment-requiring group. Results of this study showed that most nROP patients had only 1 outer retinal layer while in some tROP patient’s outer retina was fully developed (1 layer FOS, 95% in nROP vs. 53.2% with P = 0.002) (Table 2). Even in tROP patients, the development of the outer retina was delayed in btROP patients (patients who had worse and more severe condition) compared with ltROP patients (55% in btROP versus 11.5% in ltROP group had a 3-layer outer segment, p-value < 0.001). It might be explained theoretically as the alteration in normal retinal development as a result of an imbalance in retinal chemicals. As animal studies demonstrated, outer retinal development continues throughout the perinatal period [24]. We hypothesized that the oxidative stress and increased VEGF that necessitates the treatment may result in better development of the outer segment. This assumption requires further clinical and animal studies to be proven.

To the best of our knowledge, this is the first study that has reported the presence of HRF in pretreatment imaging of ROP patients. We found that 17.2% with ROP showed hyperreflective changes in pretreatment OCT imaging. The presence of HRF was not different in tROP and nROP groups (Table 2). HRF is a novel OCT biomarker with several possible explanations including migration of RPE cells, and microglia, and even could be interpreted as a precursor of intraretinal neovascularization in the clinical setting [25]. It has been postulated that this marker could predict poor response to steroid treatment in the case of diabetic macular edema [26]. However, its prevalence and utility in ROP management are ambiguous. Also, it is unclear whether HRF is a sign of foveal immaturity or it is a consequence of vascular anomaly. Further prospective studies are required to determine the role of HRF in ROP management.

Although the foveal pit shape is the most apparent sign of foveal immaturity, the qualitative description of deepening and contour irregularity was not useful for differentiating tROP from nROP (Table 2). Alterations in foveal retinal layers are also a hallmark of foveal development. Inner and outer retinal layers follow different paths of development in the macular area [13], therefore we described the presence of IRL and its thickness and also the thickness of ONL and its pattern. The result of this study exhibited that IRL extrusion was incomplete in both tROP and nROP groups. Interestingly, the presence of thicker IRL was more frequent in the nROP group in comparison with the tROP group (100% vs.64.8%, P-value = 0.001). Moreover, immature outer retina with attenuation of outer segment photoreceptor layers was more common in nROP than in tROP patients. Besides, the result of the current study didn’t reveal any difference between the presence or type of ONL widening among tROP and nROP patients. In accordance with our results, Vogel et al., in a longitudinal OCT imaging of tROP and nROP infants, showed a higher presence of IRL at the foveal center [13]. Also, Dubis et al. showed the persistence of inner retinal layers regardless of ROP severity [27]. Therefore, it seems with present-day ophthalmoscopic-based planning for ROP treatment, patients who don’t receive treatment are shown to have a more immature foveal structure compared with treatment treatment-requiring group. It is proven that an immature foveal structure (thinner ONL in the retina with a shallow foveal pit) is associated with long-term functional loss in ROP patients [5]. However, it is unclear whether alterations in the foveal maturation of ROP patients should be viewed as a coincidence due to infants’ prematurity or consequent to vascular pathologies.

To increase understanding of the pretreatment structural difference between tROP and nROP groups, we quantitatively measured several foveal indices: FPD, FPW, FPA and CFT. Other findings of this study were significantly higher FPW and FPA in tROP than in nROP (103.83 vs. 70.79 and 66.53 vs. 41.5 respectively). These parameters may be related to less maturity of the fovea. We found that GA and BW were negatively correlated with CFT (Table 3). Similar studies on infants also found a similar negative correlation between GA, BW and CFT [11, 28, 29]. Our results also showed that although mean CFT was higher in tROP than nROP and also was higher in btROP with more severe than ltROP; however, the difference didn’t reach statistical significance. On the other hand, the order from lowest to highest thickness was nROP, ltROP and btROP respectively. The statistically insignificant might be partially due to a small number of patients in each group and it requires future studies with a large number of cases. One of the explanations was lower GA and BW. It might be partially explained by more amounts of VEGF and other growth factors in vitreous patients with more severe disease. Similarly, Gursoy et al. found an insignificant difference in CFT between nROP and tROP groups [10]. An increase in CFT could be considered a sign of foveal immaturity as previous studies comparing ROP patients with term infants illustrated higher CFT in ROP patients [10, 30]. This difference continues to be evident even years after, in studies on children and younger adults with a history of ROP and may contribute to loss of visual function in these patients [29, 31]. Moreover, a report of comparison between LPC and bevacizumab-treated patients shows differences in CFT of follow-up OCT imaging [23, 32]. However, we demonstrated the difference in pre-treatment of ltROP and btROP was also insignificant (Table 2). Therefore, the change in post-treatment values should be regarded as a result of the treatment itself not the stage of ROP.

For the first time, we developed a regression model to anticipate the need for treatment based on macular OCT findings. We found that by measuring FPD and CFT in addition to GA, it is possible to predict treatment requirements with sensitivity, specificity and diagnosis accuracy of 97%, 65% and 91.7% respectively.In a study on children and younger adults with a history of spontaneously regressed ROP and treat ROP patients, Akula et al. measured FPD, CFT and FPW from OCT imaging. They found that FPD is significantly different between these tROP and nROP groups. Also, they revealed that FPD was the only statistically significant predictor of visual acuity in their case series rather than ROP severity [22]. Moreover, recent studies have revealed that lower FPD is associated with prematurity as it was shown to be positively correlated with BW and negatively correlated with GA [33, 34]. Thus, FPD in addition to CFT could be viewed as an indicator of foveal immaturity. These indices besides the aforementioned morphological findings could be used as an axillary tool in challenging cases. Early retinal alterations detected by OCT imaging may improve visual prognosis and complement clinical diagnoses and management.

This study has several limitations. We focused on the macular retinal parameters; this does not exclude the importance of peripheral vascular parameters in ROP. Future studies should plan to evaluate these factors using OCTA devices. The procedure of using a handheld OCT for infants was time-consuming and was prone to imaging artifacts, future devices with faster imaging acquisition may enhance the quality and feasibility of image acquisition. We manually measured retinal parameters from the B-scan with the steepest foveal pit, automatically derived retinal parameters from multiple B-scans could improve precision. Finally, we have a small number of ROP infants in the no-treatment group and our patients didn’t undergo longitudinal examinations.

## Conclusion

In conclusion, we found that CME and ERM are common macular changes in OCT of ROP patients with or without indication for treatment. Foveal immaturity indices including the presence of IRL in the fovea, absence of foveal ONL widening and attenuation of hyperreflective OS layers are evident in both tROP and nROP groups and couldn’t distinguish the need for treatment. However, gestational age, the foveal pit depth and the central foveal thickness could accurately predict the need for treatment. Additional research is required to establish the potential application of these parameters in clinical practice.

## Abbreviations

ROP: Retinopathy of prematurity
SD-OCT: spectral domain optical coherence tomography
IVB: Intravitreal Bevacizumab
FPD: Foveal pit depth
FPW: foveal pit width
CFT: central foveal thickness
